# Syndrome naviculo-capital de Fenton (à propos d’un nouveau cas)

**DOI:** 10.11604/pamj.2017.26.206.9288

**Published:** 2017-04-17

**Authors:** Azzelarab Bennis, Abdellatif Benabbouha, Mohammed Reda Ouzaa, Adil Lamkhanter, Mohammed Benchakroun, Abdelouahab Jaafar

**Affiliations:** 1 Service de Traumatologie-Orthopédie, Hôpital Militaire d’Instruction Mohammed V, Université Mohammed V, Rabat, Maroc

**Keywords:** Fracture, scaphoïde, capitatum, Fracture, scaphoid, capitatum

## Abstract

La fracture scapho-capitale ou syndrome naviculo-capital de Fenton, est une lésion très rare, souvent méconnue. Elle résulte d'un traumatisme du poignet de haute énergie. Son mécanisme est controversé. Les auteurs rapportent l'observation d'un patient,qui a présenté dans les suites d’un accident de la voie publique une fracture du scaphoïde associée à une fracture du capitatum, du triquetrum et de la styloïde cubitale. La voie d'abord dorsale, a permis une réduction et une contention par embrochage de la première rangée, indépendamment de la deuxième rangée. Les ligaments interosseux scapho-lunaire et lunaro-triquétral étaient intacts. Une immobilisation plâtrée antébrachio-palmaire a été mise en place pour 12 semaines. La rééducation a durée six mois. A deux ans de recul, le score fonctionnel de Cooney était bon et l'ensemble des fractures a consolidé sans aucune désaxation intracarpienne. La reprise du travail était au huitième mois après l’accident.

Scapho-capitate fracture or Fenton’s naviculo-capitate fracture syndrome is a very rare and often ignored lesion. It is caused by a high-energy traumatic injury to the wrist. Its mechanism is controversial. This study reports the case of a patient with scaphoid fracture associated with fracture of the capitatum, triquetrum and ulnar styloid due to public road accident. Dorsal approach allowed reduction and containment by internal fixation in the first row, regardless the second row. Scapholunate interosseous ligaments and luno-triquetral were intact. The patient underwent antebrachial-palmar plaster immobilization for 12 weeks. Rehabilitation program lasted for six months. Cooney’s wrist function score was good and all fractures consolidated without intracarpal malalignment at 2 years follow up. The patient resumed work in the eighth month after the road accident.

## Introduction

La fracture scapho-capitale ou syndrome de Fenton, est une lésion rare, définie par une fracture transversale du pole proximal du capitatum, avec rotation de 180° du fragment proximal, et une fracture du scaphoïde. Le but de ce travail est de discuter les mécanismes lésionnels, les problèmes diagnostiques, et notre conduite thérapeutique à la lumière d'une revue de la littérature.

## Patient et observation

**Figure 1:**
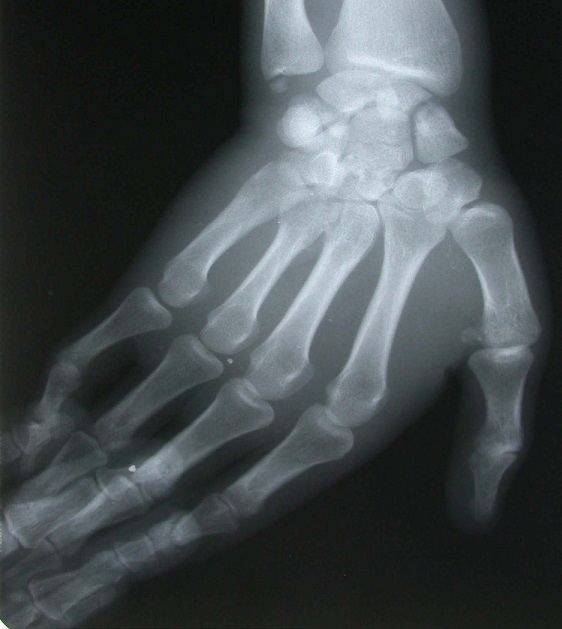
Radiographies du poignet (face-profil): fracture du scaphoïde; du capitatum; du pyramidal et de l’apophyse styloïde cubitale, sans luxation des os du carpe

**Figure 2:**
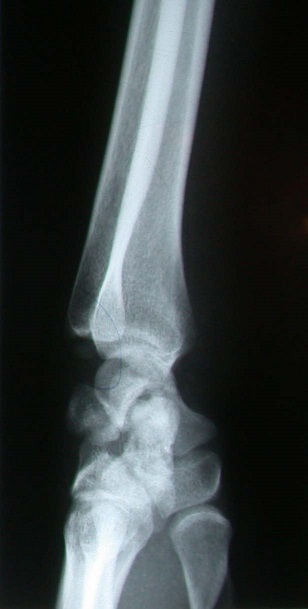
Radiographies du poignet (face-profil): fracture du scaphoïde; du capitatum; du pyramidal et de l’apophyse styloïde cubitale, sans luxation des os du carpe

**Figure 3:**
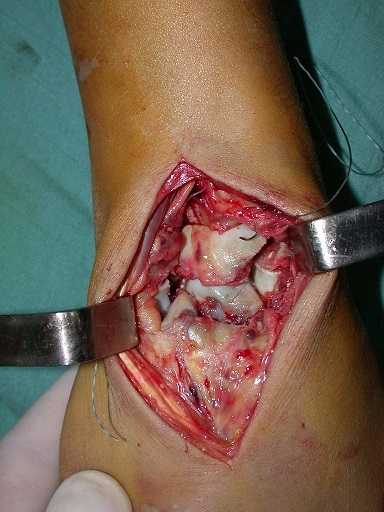
Vue préopératoire: fracture du scaphoïde; du capitatum et du pyramidal

**Figure 4:**
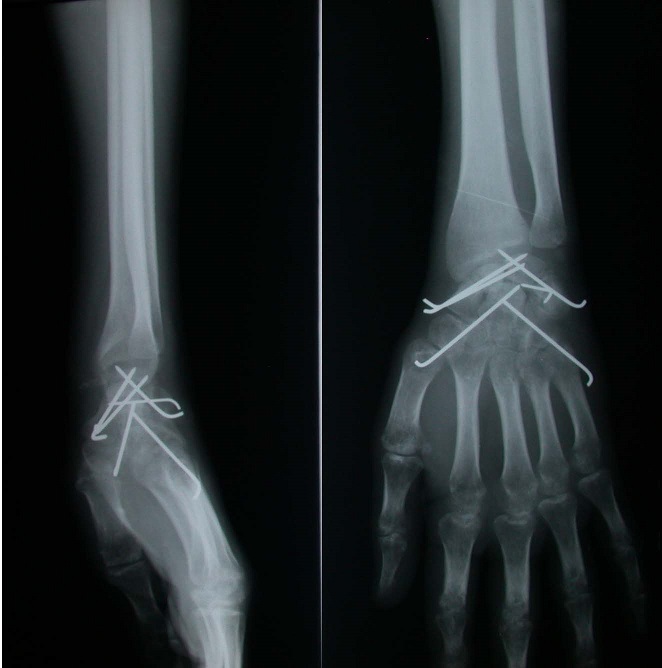
Radiographies de contrôle après embrochage des os du carpe

Il s'agit d’un patient âgé de 26 ans, droitier, travailleur manuel, ayant présenté à la suite d'un accident de la voie publique, un traumatisme du poignet droit, avec réception direct sur la paume de la main, poignet en extension. Le bilan radiologique (Figure 1, Figure 2) avait montré une fracture du scaphoïde, du capitatum, du pyramidal et de l’apophyse styloïde cubitale, sans luxation des os du carpe (lésion type 1 de Vance). Le patient a été pris en charge dans les 24 heures qui ont suivi le traumatisme. L'abord du poignet était dorsal, longitudinal, centré sur l'interligne radiocarpien, entre le troisième et le quatrième compartiment des extenseurs (Figure 3). Le nerf interosseux postérieur a été coagulé. La réduction anatomique a été ensuite réalisée manuellement sous contrôle visuel, à l'aide de broche et de spatule. L’embrochage de la première rangée des os du carpe est préparé par mise en place de deux broches croisées de Kirschner introduites depuis la facette lunarienne du scaphoïde et deux autres au niveau du triquetrum. L’ostéosynthèse du capitatuma été réalisée par embrochage croisé de la deuxième rangée, les deux broches se terminent au niveau du pole proximale du capitatum (Figure 4). Une immobilisation plâtrée antébrachio-palmaire, prenant la première phalange du pouce et maintenant le poignet en position neutre, est mise en place pour 12 semaines. Les broches sont retirées sous anesthésie locale. La rééducation a durée six mois.

**Figure 5:**
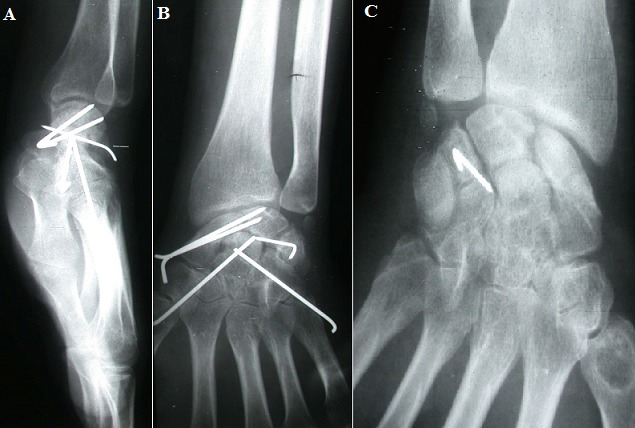
(A,B, C) radiographies du poignet (face, profil): consolidation osseuse des os du carpe

A deux ans de recul, le résultat fonctionnel était très satisfaisant.Cliniquement, le score fonctionnel de Cooney était bon: l’extension était de 60°; la flexion à 50°; la force de serrage moyenne était de 78% par rapport au côté sain. Radiologiquement, l'ensemble des fractures a consolidé et il n'existe pas de désaxation intra-carpienne en DISI ou en VISI (Figure 5). Aucune complication n’a été relevée ni dans les suites opératoires, ni à long terme. La reprise du travail était au huitième mois après l’accident.

## Discussion

La fracture scapho-capitale est une lésion rare, dont le mécanisme est controversé. En effet, pour Fenton [1], la double fracture, est due à une chute sur la main, poignet en extension et en inclinaison radiale. La styloïde radiale fracture le scaphoïde, puis l'énergie résiduelle fracture le pôle proximal du grand os, qui subit une poussée latérale responsable de la bascule dans le plan frontal. Adler [2], Marsh [3], Van Cauwenberghe [4], ont proposé des mécanismes voisins.Ce mécanisme est insuffisant pour expliquer l'association fréquente à une luxation péri-lunaire du carpe. Stein [5], a proposé un mécanisme plus satisfaisant; la chute s'effectue sur la paume de la main, le poignet en extension forcée entraîne une fracture du scaphoïde uniquement par compression, sans contact styloïde radiale-scaphoïde. Cette fracture permet l'hyperextension nécessaire au contact marge postérieure du radius et col du capitatum, responsable de la fracture du capitatum. La poursuite du mouvement entraîne une rotation de 90° du pôle proximal du capitatum fracturé, une nouvelle rotation de 90° s'effectue lors du retour en position neutre. Ces deux rotations dans le même sens entraînent un mouvement circulaire de 180° du pôle proximal du capitatum dans le plan sagittal. Johnson [6] précise que lors de l'hyper extension du poignet, si la tête du capitatum ne se luxe pas en arrière, le lunatum peut décapiter le grand os avec sa marge postérieure. Vance [7] n'élimine pas dans certains cas un mécanisme en flexion. Dans ce cas la marge antérieure du radius entrerait en contact avec le col du capitatum, après la fracture du scaphoïde.

Pour notre part, nous pensons que l’hyperextension du poignet en inclinaison cubitale, va entrainer une fracture du scaphoïde et rupture des ligaments antérieurs du carpe qui maintiennent la concavité et la stabilité de ce dernier. Le radius et le pôle proximal du scaphoïde ainsi que le semi lunaire forme un seul bloc, qui suite à l’exagération de l’hyperextension,va décapiter le capitatum par effet « tire bouchon » et va entrainer en plus une fracture du triquetrum par avulsion du ligament luno-triquetral. Le diagnostic clinique est difficile, reflété par le retard de diagnostic qui est un phénomène très fréquent [8] et ce d'autant plus qu'il n'existe pas de luxation associée. La fracture du scaphoïde est habituellement diagnostiquée, mais la lésion du capitatum passe inaperçue, le diagnostic est alors fait à distance lors des contrôles. Il est donc nécessaire devant toute fracture du scaphoïde, d’accorder une attention toute particulière aux images radiologiques du capitatum et du triquetrum. La clinique recherchera en plus une lésion nerveuse associée (atteinte du nerf médian).

La radiographie standard de face et de profil permet d'affirmer le diagnostic. Le cliché de profil permet de classer les lésions selon les critères de Vance [7]: type 1: le carpe est aligné, sans aucun élément luxé; Type 2: luxation dorsale du carpe, emportant le pole proximal du capitatum; Type 3: luxation dorsale du carpe, le pole proximal du capitatum restant dans la concavité du semi-lunaire; Type 4: seul le pôle proximal du capitatum est luxé dorsalement; Type 5: luxation palmaire du carpe avec le pôle proximal du capitatum; Type 6: luxation palmaire isolée du pôle proximal du capitatum.

Notre patient avait une lésion de type 1 avec un carpe aligné, sans aucun élément luxé; cettelésion est moins fréquente que les types 2 et 3. Les trois premiers types représentent la majeure partie des fractures scapho-capitales. Vance ne retrouve qu'un seul cas des types 4, 5, et 6 dans la littérature.

L'analyse très soigneusement des radiographies de face du poignet a objectivé des fractures du grand os et du pyramidal dans notre cas. Parfois le scanner est nécessaire pour compléter le bilan. La luxation des os du carpe est souvent associée à la fracture scapho-capitale. C'est bien entendu un élément aggravant le tableau et un facteur de compression du nerf médian [8].

Si le traitement chirurgical ne paraît pas se discuter, la voie d'abord doit être choisie en fonction des lésions anatomiques et du respect de l'anatomie vasculaire. La voie d'abord antérieure est logique en cas d'atteinte du nerf médian (décompression nerveuse par ouverture du canal carpien) ou en cas de lésions ligamentaires (réparation ligamentaire).Cette voie antérieure préserve la vascularisation des os du carpe, évitant la pseudo darthrose et la nécrose de ces derniers, cependant la réduction des fractures est très laborieuse en raison du jour limité.

Dans les autres cas, la voie postérieure est la plus utilisée, elle doit rester médiane pour éviter tout risque de nécrose du scaphoïde. La vascularisation du capitatum est distale selon Razemont [9], ce qui explique le peu de risque d'atteinte lors d'une luxation, ou lors d'un abord si celui ci reste proximal. Par contre le scaphoïde est vascularisé en dorsal, avec des orifices de pénétration sur tout le tiers distal selon Watson [10], entraînant un plus grand risque lors de l'abord postérieur et des manœuvres de réduction et d'ostéosynthèse. La voie postérieure permet de réduire la luxation du carpe et la bascule du pôle proximal du capitatum, chose qui est impossible par manoeuvres externes [11]. Elle contrôle l'ensemble des lésions osseuses carpiennes dans la majorité des cas [12-14]. Un abord palmaire complémentaire peut être utile en cas d'irréductibilité par la seule voie dorsale. De rares auteurs ont préconisé une voie d'abord uniquement palmaire, notamment pour les formes trans-scapho-périlunaires [15]. La tendance actuelle est à la voie d'abord combinée palmaire et dorsale systématique [16-18].

Dans notre cas, nous avons opté pour une voie d’abord dorsale unique en absence de luxation intra-carpienne. Elle nous a permis une réduction et une contention par embrochage des os de la première rangée, indépendamment de ceux de la deuxième rangée. Dans les formes associées à une luxation périlunaire, la réduction de la luxation par des manœuvres externes est déconseillée, l'abord chirurgical est systématique. La résection de la première rangée du carpe en urgence n’est envisageable, qu’en cas d'exérèse du pôle proximal du scaphoïde, rendue nécessaire par le risque élevé de nécrose, lorsque le fragment osseux se trouve complètement libre à la face postérieure du radius. Cette résection du pôle proximal du scaphoïde déstabilise le carpe et incite à faire une résection de la première rangée du carpe et à maintenir la hauteur carpienne par fixateur externe en distraction, elle peut êtreréalisé en un seul temps avec ostéosynthèse du capitatum ou en deuxtemps [19,20]. Le premier temps est la synthèse du capitatum du faite de l'excellente vascularisation de la partie distale du capitatum comme le préconisait Weseley [20] pour obtenir un néocondyle carpien de bonne qualité (la tête du capitatum doit être indemne de toute lésion cartilagineuse), puis un deuxième temps de résection de la première rangée.

## Conclusion

Bien qu'exceptionnelle, le syndrome naviculo-capital mérite d'être bien connue en raison de ses particularités radiologiques et thérapeutiques. Il est souvent associé à une luxation du carpe, le diagnostic positif repose sur la radiographie standard, dont l'interprétation est délicate, à l’origine de retard de diagnostic ou de diagnostic incomplet. Devant toute luxation trans-scapho-périlunaire ou fracture du scaphoïde, on doit rechercher une fracture du capitatum et du triquetrum. La prise en charge précoce et la réduction anatomique parfaite des lésions sont les seuls garants d’un bon résultat fonctionnel. Le traitement est chirurgical, imposant la voie d'abord la moins agressive possible, en fonction de la lésion elle-même et des lésions associées.

## References

[1] Fenton RL (1956). The naviculo-capitate fracture syndrome. J Bone Joint Surg Am.

[2] Adler JB, Shaftan GW (1962). Fractures of the Capitate. J Bone Joint Surg Am.

[3] Marsh AP, Lampros PJ (1959). The naviculo-capitatefracture syndrome. Am JRoentgenol Radium The Nucl Med.

[4] Van Cauwenberghe R (1957). Rare case of carpal fracture-dislocation. Acta Orthop Belgica.

[5] Stein F, Siegel MW (1969). Naviculo-capitate fracture syndrome. J Bone Joint Surg Am.

[6] Johnson RP (1980). The acutely injured wrist and its residuals. Clin Orthop Relat Res.

[7] Vance RM, Gelberman RH, Evans EF (1980). Scaphocapitatefractures - patterns of dislocation, mechanisms of injury, andpreliminary results of treatment. J Bone Joint Surg Am.

[8] Boisgard S, Bremont JL, Guyonnet G, Chantenet T, Levai JP (1996). Scapho-capitate fracture: a propos of a case, review of the litera¬ture. Ann Chir Main Memb Super.

[9] Razemon JP (1984). Fractures des os du carpe. Traité de Chirurgie de la Main T 2.

[10] Watson J (1955). Fractures and joint injuries.

[11] Alnot JY, Houvet P (1995). Chirurgie des traumatismes récents du carpe. EMC. Tech Chir.

[12] Adkison JW, Chapman MW (1982). Treatment of acute lunate and perilunate dislocations. Clin Orthop Relat Res.

[13] Mayfield JK, Johnson RP, Kilcoyne RK (1980). Carpal dislocations: pathomechanics and progressive perilunar instability. J Hand Surg Am.

[14] Campbell RD, Thompson TC, Lance EM, Adler JB (1965). Indications for openreduction of lunate and perilunate dislocations of the carpal bones. J BoneJoint SurgAm.

[15] Viegas SF, Bean JW, Schram RA (1987). Transscaphoid fracture/dislocations treated with open reduction and Herbert screw internal fixation. J Hand Surg Am.

[16] Apergis E, Maris J, Theodoratos G, Pavlakis D, Antoniou N (1997). Perilunate dislocations and fracture-dislocations - Closed and early open reduc¬tion compared in 28 cases. Acta Orthop Scand.

[17] Hildebrand KA, Ross DC, Patterson SD, Roth JH, MacDermid JC, King GJ (2000). Dorsal perilunate dislocations and fracture-dislocations: question-naire, clinical, and radiographic evaluation. J Hand SurgAm.

[18] Kozin SH (1998). Perilunate injuries: Diagnosis and treatment. J Am Acad Orthop Surg.

[19] Mestdagh H, Bailleul J-P, Lakaki A, Moulront S, Vilette B (1983). Vascularisationartérielle du poignet. Monogra¬phie du GEM n 12. Expansion Scientifique Française.

[20] Weseley MS, Barenfeld PA (1972). Trans-scaphoid, transcapitate transtriquetral, perilunate fracture-dislocation of the wrist. J Bone Joint Surg.

